# Polymorphic variants in exon 8 at the 3' UTR of the *HLA-G *gene are associated with septic shock in critically ill patients

**DOI:** 10.1186/cc11845

**Published:** 2012-10-29

**Authors:** Pietra Graebin, Tiago D Veit, Clarice S Alho, Fernando S Dias, José AB Chies

**Affiliations:** 1 Laboratory of Immunogenetics of Federal University of Rio Grande do Sul - 9500, Bento Gonçalves Av., Building 43323//Room 212, Porto Alegre, RS, Brazil; 2 Laboratory of Human and Molecular Genetics of Pontifical Catholic University of Rio Grande do Sul, 6681, Ipiranga Av., Building 12C, Porto Alegre, RS, Brazil; 3 São Lucas Hospital, Pontifical Catholic University of Rio Grande do Sul, 6681, Ipiranga Av., Building 12C, Porto Alegre, RS, Brazil

## Abstract

**Introduction:**

Critically ill patients are characterized as individuals hospitalized in the Intensive Care Unit (ICU) and can evolve to sepsis, septic shock or even death. Among others, genetic factors can influence the outcome of critically ill patients. HLA-G is a non-classical class Ib molecule that has limited protein variability, presenting seven isoforms generated by alternative splicing, and presents immunomodulatory properties. Polymorphisms at the 3'UTR are thought to influence *HLA-G *gene expression. It was previously observed that increased sHLA-G5 levels were predictive of survival among septic shock patients. We assessed the frequencies of 7 polymorphisms in exon 8 at the 3' UTR of *HLA-G *and associated these variants with different clinical outcomes in critically ill patients.

**Methods:**

Exon 8 at the 3' UTR of the *HLA-G *gene from 638 critically ill subjects was amplified by PCR and sequenced. Genotypes were identified using FinchTV software v.1.4.0 and the most probable haplotype constitution of each sample was determined by PHASE software v.2.1. Haplotype frequencies, linkage disequilibrium, heterozygosity test and Hardy-Weinberg Equilibrium were estimated using ARLEQUIN software v.3.5.

**Results:**

Among all critically ill patients, an association between carriers of the +2960IN_+3142 G_+3187A haplotype and septic shock (P = 0.047) was observed. Septic patients who carried the +2960IN_+3142G_+3187A haplotype presented an increased risk for septic shock (P = 0.031).

**Conclusions:**

The present study showed, for the first time, an association between polymorphisms in exon 8 at the 3 'UTR of *HLA-G *gene and outcomes of critically ill patients. These results may be important for understanding the mechanisms involved in evolution to septic shock in critically ill patients.

## Introduction

Critically ill patients are individuals hospitalized in the ICU, who can evolve to sepsis, septic shock, death, or survival [1]. Massive resources have been invested in sepsis control, either in public or private sectors, and sepsis is the major cause of death in Brazilian ICUs according to ILAS (Instituto Latino Americano de Sepse) [2]. Several factors influence the outcome of critically ill patients, including different degrees of inflammatory response to infections and genetic factors [3]. Human leucocyte antigen G (HLA-G) is a non-classical class Ib molecule that differs from classical class I molecules mainly due to limited protein variability, expression of seven isoforms (HLA-G1-4 membrane-bound forms and HLA-G5-7 soluble forms) generated by alternative splicing [4], and by its immunomodulatory properties [5]. HLA-G molecules, through binding to specific receptors (ILT-2 (LILRB1 and CD85j)) [6], (LT-4 (LILRB2 and CD85d)) [7] and KIR2DL4 (CD158d) [8]) can inhibit the cytotoxic activity, and mediate apoptosis of CD8+ T cells and natural killer (NK) cells, the proliferative response of CD4+ T cells, and target them for an immunosuppressive profile and the differentiation of antigen-presenting cells and B cells [9], as well as being involved in Th1/Th2 balance [10,11].

In non-pathological conditions, HLA-G expression is highly tissue-specific and can be detected in trophoblast cells, medullary cells of the thymus, cornea, pancreatic islets and adult endothelial cell precursors [12]. The HLA-G molecule has been implicated in several conditions, such as pregnancy [13-15], acceptance of allografts [16-18], evasion of tumors [19-24] or viral infections [25-32] from immune response, inflammatory autoimmune diseases [33-47], and spontaneous intracerebral hemorrhage [48]. So far, only one study has investigated HLA-G expression in critically ill patients with septic shock, observing that increased HLA-G5 levels were predictive of survival among these patients [49].

The *HLA-G *gene is located in the HLA complex at the 6p21.3 chromosome region [50,51]. *HLA-G *gene expression is regulated by several factors which are not yet completely elucidated, and both its long promoter region, and its 3' untranslated region (UTR) are supposed to play important roles in this process. Of note, the 3' UTR contains eight polymorphisms, most of which are localized at putative miRNA binding sites [52,53], and three of them were previously associated with differential expression levels of HLA-G: a 14 bp insertion/deletion at position +2960 (rs1704), which is also associated with alternative splicing [54]; a C/G SNP at +3142 (rs1063320), which is located within an miRNA target site and has been shown to differentially affect miRNA binding [52,53,55]; and an A/G SNP at +3187 (rs9380142) that is found 4-bp upstream, an AUUUA motif which has been recognized as an (AU)-rich motif sequence that mediates mRNA degradation [56]. The 14 bp insertion has been repeatedly associated with lower levels of the molecule in different studies [37,57-60]. It is noteworthy that this variant is almost always accompanied by +3142G and +3187A alleles, both previously associated with low mRNA availability, indicating that the lower mRNA production associated with 14-bp insertion might be a consequence of the combined presence of these polymorphisms [61].

In the present study, we assessed the frequencies of the haplotype composed by the 14 bp insertion, the +3142G and the +3187G SNPs and, additionally, the allelic and genotypic of the above-mentioned polymorphisms and four other 3'UTR *HLA-G *SNPs: +3003C>T (rs1707), +3010C>G (rs1710), +3027A>C (rs17179101) and +3035C>T (rs17179108), and sought to associate these variants with different clinical outcomes in critically ill patients.

## Materials and methods

### Patients

Blood samples were obtained from 638 ICU patients from Hospital São Lucas of Pontifícia Universidade Católica do Rio Grande do Sul (PUCRS), located in Porto Alegre, Rio Grande do Sul, the southernmost state of Brazil, between 2004 and 2010. Definition of sepsis and septic shock followed the American College of Chest Physicians/Society of Critical Care Medicine Consensus Conference Committee criteria [62]. Sepsis is defined as the occurrence of systemic inflammatory response syndrome (SIRS) caused by the presence of infection or clinical evidence of infection. SIRS is manifested by two or more of the following symptoms: fever (temperature > 38.0°C) or hypothermia (temperature < 35.5°C), tachycardia (> 90 beats/minute), tachypnea (> 20 breaths/minute), leukocytosis or leukopenia (white blood cell count > 12.000 or < 4.000 cells/µl). Septic shock is defined as the presence of SIRS, documented infection or positive blood culture or organ dysfunction, hypoperfusion abnormality or hypotension. The definition of hypotension is systolic blood pressure < 90 mm Hg or a reduction in systolic blood pressure of 40 mm Hg.

The following exclusion criteria were applied for this study: 1 HIV-positive patients, 2) pregnancy, and 3) patients receiving immunosuppressive therapy. Also, patients readmitted to ICU from whom data were previously collected were excluded. Social and demographic data (age, sex, length of stay in ICU and in hospital) and clinical data (dysfunction and failure of six organ systems during the first week of ICU admission (Sequential Organ Failure Assessment, SOFA score), and physiological variables on the day of ICU admission (Acute Physiology and Chronic Health Evaluation, APACHE II score) were obtained from all patients, in addition to the occurrence of sepsis, septic shock and death.

The protocol of the study was approved by the PUCRS Ethics Committee (CEP-11/05593) and all patients or their surrogates gave written informed consent before blood withdrawal.

### 3' UTR polymorphisms evaluation

Genomic DNA was extracted according to Lahiri and Nurnberger (1991) [63]. Exon 8 at the 3' UTR of the *HLA-G *gene of each sample was amplified by PCR (primers HLA-G8F: 5' TGTGAAACAGCTGCCCTGTGT3' and HLA-G8R: 5'GTCTTCCATTTATTTTGTCTCT 3') [61]. Amplification was performed in a final volume of 25 µl containing 0.2 mM each of dNTP; 1.5 mM MgCl_2_; 10 pmol/µl of each primer; 1 unit of Taq Platinum High Fidelity in 1X PCR-specific buffer (Invitrogen-Life Technologies, São Paulo, Brazil) and 10 to 100 ng of genomic DNA. The initial denaturation cycle was carried out at 94ºC for 5 minutes; followed by 35 cycles at 95ºC for 45 s; 56ºC for 45 s; 72ºC for 1 minute and by a final extension step at 72ºC for 7 minutes. The amplification product was quantified (10 to 50 ng/µl) by Low Mass (Invitogen-Life Technologies, São Paulo, Brazil) in 1% agarose gel. Fragments with 14 bp insertion presented 361 pb. PCR products were directly sequenced using the primer HLA-G8R: 5'GTCTTCCATTTATTTTGTCTCT 3' in an ABI 3730 XL DNA Sequencer according to the manufacturer's manual. Genotyping was performed by interpretation of chromatogram peaks by sequencing FinchTV software version 1.4.0.

The PHASE software version 2.1 [64,65] was used to determinate the most probable haplotype constitution of each sample. Six independent runs with different seed values provided the same results by PHASE methods. The lowest probability value was between 0.9650 and 1.0000. Haplotype frequencies, linkage disequilibrium (LD), heterozygosity test and Hardy-Weinberg Equilibrium (HWE) were defined using ARLEQUIN software version 3.5 [66].

### Statistical analysis

Statistical calculations were performed using statistical package SPSS 18 (SPSS 18.0 for Windows, Chicago, Illinois, USA). Unless otherwise stated, continuous variable results were expressed as mean ± SD, and categorical variables as frequencies and percents. For the categorical data we used Pearson chi square test. The Bonferroni correction was applied when *P *values were significant. All reported *P*-values are two-tailed and considered statistically significant when 0.05 or less.

## Results and discussion

The median age among the 638 critically ill patients (53.0% male and 47.0% female) was 57 years (13_min_/97_max_). Of the 638 patients, 73.5% (n = 469) developed sepsis, 51.6% (n = 329) progressed to septic shock, 32.9% (n = 210) died in the ICU; the mortality rate in the ICU + hospital was 45.7% (n = 290). Other clinical details are displayed in Table 1.

**Table 1:** Clinical and demographic data of critically ill patients

Variables	Values in critically ill patients (n = 683)
Age, years	57 (13/97)
Male, n (%)	338 (53.0)
APACHE II score, mean (SD)	19.32 (7.73)
SOFA scores, median (min/max)	
SOFA-1	6 (0/18)
SOFA-2	6 (0/17)
SOFA-3	6 (0/18)
SOFA-4	6 (0/22)
SOFA-5	5 (0/20)
SOFA-6	5 (0/21)
SOFA-7	5 (0/24)
SOFA-15	5 (0/19)
SOFA-29	5 (0/16)
Length of stay, days, median (min/max)	
ICU	13 (0/173)
ICU + hospital	35 (1/242)
With sepsis, n (%)	469 (73.5)
With septic shock, n (%)	329 (51.6)
ICU mortality, n (%)	210 (32.9)
ICU + hospital mortality, n (%)	290 (45.7)

APACHE-II: Acute Physiology and Chronic Health Evaluation II; SOFA: Sequential Organ Failure Assessment; n: number of patients.

### **Allelic, genotype, haplotype and diplotype frequencies**

Table 2 presents allelic and genotypic frequencies of polymorphisms in critically ill patients. These frequencies are in agreement with other studies in Brazilian populations with a similar ethnic background [67,68]. Therefore, our sample is representative of the population of southern Brazil, which has a major contribution from European ancestry [68,69].

**Table 2:** HLA-G 3'UTR allelic and genotypic frequencies among critically ill patients

	+2960 INDEL		+3003C>T		+3010C>G		+3027A>C		+3035C>T		+3142C>G		+3187A>C	
**Allelic**	DEL	0.578	C	0.109	C	0.521	A	0.034	C	0.861	C	0.463	A	0.699
	IN	0.421	T	0.890	G	0.478	C	0.965	T	0.141	G	0.536	C	0.304
**Genotypic**	DEL/DEL	0.310	C/C	0.013	C/C	0.251	A/A	0.001	C/C	0.734	C/C	0.180	A/A	0.448
	DEL/IN	0.536	C/T	0.194	C/G	0.541	A/C	0.066	C/T	0.255	C/G	0.566	A/C	0.503
	IN/IN	0.154	T/T	0.793	G/G	0.208	C/C	0.933	T/T	0.011	G/G	0.254	C/C	0.049
***P *_HWE_**		>0.05		<0.05		>0.05		<0.05		<0.05		>0.05		>0.05

HWE: Hardy-Weinberg equilibrium.

The heterozygosity test indicated an observed heterozygosity higher than expected at the +2960INDEL, +3010C>G, +3142C>G and +3187A>G polymorphisms (Table 3). These polymorphisms were not in HWE. Castelli *et al*. (2011) also reported disagreement on HWE for +2960INDEL polymorphism in a Brazilian population. It is interesting that the diversity of the *HLA-G *locus is eight times higher than the average of the diversity of the human genome [55]. Moreover, among the *HLA-G *regions, the 3' UTR region shows more diversity than the promoter and the 5' upstream regulatory regions (5'URR) [67].

**Table 3:** Heterozygosity test

Polymorphism	Observed	Expected
+2960INDEL*	0.53605	0.4881
+3003C>T	0.19436	0.19551
+3010C>G*	0.54075	0.4995
+3027A>C	0.06583	0.06664
+3035C>T	0.25549	0.23913
+3142C>G*	0.56583	0.49768
+3187A>G*	0.5047	0.42108
Mean	0.38043	0.34395
SD	0.20369	0.17535

*Polymorphisms with observed heterozygosity values greater than expected.

A strong LD was observed between all polymorphic sites (*P *< 0.01, data not shown), which is consistent with data available in the literature [61]. According to the PHASE software, there were 22 different inferred haplotypes among critically ill patients. The most frequent haplotypes were UTR-1 (27.90%), UTR-2 (26.65%), UTR-3 (10.42%), UTR-5 (10.03%) and UTR-4 (8.93%) (haplotype nomenclature according to [61]). The two most frequent haplotypes observed among critically ill patients were also the most frequent in populational studies with southern Brazilian healthy populations [61,67,68]. Also, we observed 64 different diplotypes, with the DTGCCCG/ITCCCGA (UTR-1/UTR-2) diplotype being the most frequent among critically ill patients (15.8%). Assuming that the Brazilian population is a heterogeneous one [67], this diversity was expected due to its colonization history [68,69]. Furthermore, it has already been suggested, and our data also support this view, that this region is under balancing selection, in which there is a selection of heterozygotes [55,67]. The selection of heterozygote could be evolutionarily advantageous, since it could promote a balance between high and low HLA-G expression, according to the biological context.

### Polymorphic variants of HLA-G and outcomes of critically ill patients

Possible outcomes considered were sepsis, septic shock, ICU mortality and ICU+hospital mortality among patients with septic shock. The genotypes of each SNP were tested separately for progression to sepsis, septic shock, death in the ICU and death in the ICU + hospital; however no significant differences were observed. Haplotype and diplotype frequencies, when subjected to the same tests, were not associated with the considered outcomes (data not shown).

From all analyzed variants, +2960IN, +3142G and +3187A alleles had already been associated with reduced HLA-G expression [57,61]. Considering a potential interaction effect and the strong LD observed for these three variants, haplotypes encompassing those variants were determined and the presence/absence of this particular haplotype (+2960IN_+3142G_+3187A) was analyzed in relation to the studied clinical outcomes.

As can be observed in Table 4, among all critically ill patients, carriers of the +2960IN_+3142G_+3187A haplotype were more likely to develop septic shock (*P *= 0.047). When considering only patients who developed sepsis, carriers of this haplotype were more likely to develop septic shock (odds ratio, OR = 1.62, 95% CI 1.04, 2.50, *P *= 0.031) (Table 5). When analyzing mortality among patients with septic shock, no significant differences were observed (Table 5).

**Table 4:** Carriers and noncarriers of the +2960IN_+3142G_+3187A haplotype and clinical features among 683 critically ill patients

Variables	Number (%) of carriers of +2960IN_+3142G_+3187A	Number (%) of noncarriers of +2960IN_+3142G_+3187A	*P*-value
Frequency	436 (68.3)	202 (31.7)	-
With sepsis	323 (74.1)	146 (72.3)	0.701
With septic shock	237 (54.4)	92 (45.5)	0.047*
ICU mortality	149 (34.2)	61 (30.2)	0.366
ICU + hospital mortality	201 (46.1)	89 (44.1)	0.692

*P*-value, Pearson chi-Square test with the Yates correction; *comparison between carriers and noncarriers of the haplotype +2960IN_+3142G_+3187A.

**Table 5:** Presence/absence of the +2960IN_+3142G_+3187A (IN-G-A) haplotype in relation to progression to septic shock (SS) and mortality after SS among patients with sepsis

	With IN-G-An (%)	Without IN-G-An (%)	Odds ratio(95% CI)	*P*-value
With sepsis	323 (68.9)	146 (31.1)		-
Progression to SS	237 (73.4)	92 (63.0)	1.62 (1.04-2.50)	0.031*
Without SS	86 (26.6)	54 (37.0)		
ICU mortality (among SS patients)	122/237 (51.5)	48/92 (52.2)	0.97 (0.58-1.62)	0.990
ICU + hospital mortality (among SS patients)	149/237 (62.9)	61/92 (66.3)	0.86 (0.50-1.47)	0.649

*P*-value, Pearson chi-Square test with the Yates correction; *chi-square test significant (*P *< 0.05).

Next, we sought to investigate if specific clinical outcomes could be associated with a particular polymorphism among critically ill patients. For each polymorphism, individuals were grouped according to presence/absence of a given allele and tested for association between a particular allele carrier and outcomes (progression to sepsis, septic shock, ICU mortality and ICU+hospital mortality). Among all critically ill patients there were no significant differences for sepsis (Table 6).

**Table 6:** Presence/absence of each allele of a given polymorphism and sepsis in all critically ill patients

Polymorphism	Outcome	With IN, n (%)	Without IN, n (%)	*P*-value
+ 2960INDEL	Total of ICU patients	440 (69.0)	198 (31.0)	**-**
	With sepsis	326 (74.1)	143 (72.2)	0.691
	Without sepsis	114 (25.9)	55 (27.7)	

**Polymorphism**	**Outcome**	**With +3003C, n (%)**	**Without +3003C n, (%)**	***P*-value**

+3003C>T	Total of ICU patients	132 (20.7)	506 (79.3)	**-**
	With sepsis	98 (74.2)	371 (73.3)	0.831
	Without sepsis	34 (25.8)	135 (26.7)	

**Polymorphism**	**Outcome**	**With +3010C n, (%)**	**Without +3010C, n (%)**	***P*-value**

+3010C>G	Total of ICU patients	505 (79.2)	133 (20.8)	**-**
	With sepsis	373 (73.9)	96 (72.2)	0.699
	Without sepsis	132 (26.1)	37 (27.8)	

**Polymorphism**	**Outcome**	**With +3027A, n (%)**	**Without +3027A, n (%)**	***P*-value**

+3027A>C	Total of ICU patients	43 (6.7)	595 (93.3)	**-**
	With sepsis	36 (83.7)	433 (72.8)	0.116
	Without sepsis	7 (16.3)	162 (27.2)	

**Polymorphism**	**Outcome**	**With +3035T, n (%)**	**Without +3035T, n (%)**	***P*-value**

+3035C>T	Total of ICU patients	170 (26.6)	468 (73.4)	**-**
	With sepsis	125 (73.5)	344 (73.5)	0.995
	Without sepsis	45 (26.5)	124 (26.5)	

**Polymorphism**	**Outcome**	**With +3142G, n (%)**	**Without +3142G, n (%)**	***P*-value**

+3142C>G	Total of ICU patients	523 (82.0)	115 (18.0)	**-**
	With sepsis	391 (75.0)	78 (67.8)	0.159
	Without sepsis	132 (25.0)	37 (32.2)	

**Polymorphism**	**Outcome**	**With +3187A, n (%)**	**Without +3187A, n (%)**	***P*-value**

+3187A>G	Total of ICU patients	607 (95.1)	31 (4.9)	**-**
	With sepsis	447 (73.6)	22 (71.0)	0.742
	Without sepsis	160 (26.4)	9 (29.0)	

*P*-value, chi square test; *low values of *P*.

Among patients who developed sepsis, +2960IN allele carriers presented a higher frequency among patients who progressed to septic shock (*P *= 0.046), although this finding was not statistically significant after Bonferroni correction for multiple comparisons (Table 7). Among patients who developed septic shock, no significant differences in mortality rate were associated with presence/absence of a given allele (Table 7).

**Table 7:** Progression to septic shock (SS) and mortality after septic shock among patients with sepsis

Polymorphism	Outcome	With IN, n (%)	Without IN, n (%)	*P*-value	*p*_corr_
+2960INDEL	With sepsis	363 (74.4)	153 (72.9)	-	-
	Progression to SS	266/363 (73.3)	98/153 (64.1)	0.046	0.414
	ICU mortality	135/266 (50.8)	51/98 (52.0)	0.920	-
	ICU + hospital mortality	162/266 (60.9)	65/98 (66.3)	0.409	-

**Polymorphism**	**Outcome**	**With +3003C, n (%)**	**Without +3003C, n (%)**	***P*-value**	

+3003C>T	With sepsis	98 (20.9)	371 (79.1)	-	
	Progression to SS	71/98 (72.4)	258/371 (69.5)	0.663	-
	ICU mortality	44/71 (62.0)	126/258 (48.8)	0.068	-
	ICU + hospital mortality	50/71 (70.4)	160/258 (62.3)	0.244	-

**Polymorphism**	**Outcome**	**With +3010C, n (%)**	**Without +3010C, n (%)**	***P*-value**	

+3010C>G	With sepsis	373 (79.5)	96 (20.5)	-	-
	Progression to SS	267/373 (71.6)	62/96 (64.6)	0.226	-
	ICU mortality	135/267 (50.6)	35/62 (56.5)	0.487	-
	ICU + hospital mortality	166/267 (62.2)	44/62 (71.0)	0.249	-

**Polymorphism**	**Outcome**	**With +3027A, n (%)**	**Without +3027A, n (%)**	***P*-value**	

+3027 A>C	With sepsis	36 (7.7)	433 (92.3)	-	-
	Progression to SS	25/36 (69.4)	304/433 (70.0)	1.000	-
	ICU mortality	15/25 (60.0)	155/304 (51.0)	0.510	-
	ICU + hospital mortality	17/25 (68.0)	193/304 (63.5)	0.814	-

**Polymorphism**	**Outcome**	**With +3035T, n (%)**	**Without +3035T, n (%)**	***P*-value**	

+3035C>T	With sepsis	125 (26.7)	344 (73.3)	-	-
	Progression to SS	96/125 (76.8)	233/344 (67.7)	0.075	-
	ICU mortality	51/96 (53.1)	119/233 (51.1)	0.828	-
	ICU + hospital mortality	63/96 (65.6)	147/233 (63.1)	0.757	-

**Polymorphism**	**Outcome**	**With +3142G, n (%)**	**Without +3142G, n (%)**	***P*-value**	

+3142C>G	With sepsis	431 (83.5)	85 (16.5)	-	-
	Progression to SS	309/431 (71.7)	55/85 (64.7)	0.245	-
	ICU mortality	157/309 (50.8)	29/55 (52.7)	0.908	-
	ICU + hospital mortality	188/309 (60.8)	39/55 (70.9)	0.204	-

**Polymorphism**	**Outcome**	**With +3187ª, n (%)**	**Without +3187ª, n (%)**	***P*-value**	

+3187A>G	With sepsis	447 (95.3)	22 (4.7)	-	-
	Progression to SS	316/447 (70.7)	13/22 (59.1)	0.356	-
	ICU mortality	162/316 (51.3)	8/13 (61.5)	0.658	-
	ICU + hospital mortality	200/316 (63.3)	10/13 (76.9)	0.479	-

*P*-value, chi square test; *P*^corr^, *P*-value after Bonferroni correction (n = 9).

The present study observed an association between haplotypes in exon 8 at 3' UTR of the *HLA-G *gene and outcomes in critically ill patients. These results may be important for understanding the mechanisms involved on evolution to septic shock in critically ill patients. So far, there has only been one study of HLA-G in critically ill patients, in which a higher concentration of soluble HLA-G5 was observed, and was predictive of survival after progression to septic shock [49]. Our results suggest that critically ill patients who are carriers of +2960IN_+3142G_+3187A haplotype, associated with a reduced HLA-G expression, are more likely to develop septic shock. Therefore, our results reinforce the idea that decreased production of HLA-G in ICU patients could have a negative impact on patient outcome.

## Conclusions

The present study shows for the first time an association between the +2960IN_3142G_3187A haplotype and septic shock among critically ill patients. Given the immunomodulatory properties of HLA-G, we can propose an important role for HLA-G in the mechanisms that define outcomes in critically ill patients.

Opportunely, it should be interesting to analyze other parameters to confirm this association, such as Il-10 and glucocorticoids levels, and cytokine profile in critically ill patients. All these results will provide insights into the understanding of critical illness.

## Key message

• Critically ill patients and critically ill patients who evolve to sepsis carriers of the +2960IN_+3142G_+3187A haplotype, are more likely to develop septic shock.

## Abbreviations

APACHE: Acute Physiology and Chronic Health Evaluation; bp: base pair; HLA-G: human leukocyte antigen G; HWE: Hardy-Weinberg equilibrium; ILAS: Instituto Latino Americano de Sepse; LD: linkage disequilibrium; PCR: polymerase chain reaction; PUCRS: Pontifícia Universidade Católica do Rio Grande do Sul; SOFA: Sequential Organ Failure Assessment; SNP: single nucleotide polymorphism; SIRS: systemic inflammatory response syndrome; 3' UTR: untranslated region 3'.

## Competing interests

The authors declare that they have no competing interests.

## Authors' contributions

PG, TDV, CSA, FSD and JABC contributed to the study concept and design, acquisition of data, statistical analysis, interpretation of data and drafting the manuscript. FSD and CSA screened for patients. All authors read and approved the final manuscript.
